# The Roles of Neuropeptide Y (*Npy*) and Peptide YY (*Pyy*) in Teleost Food Intake: A Mini Review

**DOI:** 10.3390/life11060547

**Published:** 2021-06-10

**Authors:** Daniel Assan, Umar Farouk Mustapha, Huapu Chen, Zhiyuan Li, Yuhao Peng, Guangli Li

**Affiliations:** 1 Fisheries College, Guangdong Ocean University, Zhanjiang 524088, China; danielassan95@gmail.com (D.A.); umarfk.gh@gmail.com (U.F.M.); chenhp@gdou.edu.cn (H.C.); g_yl903@163.com (Z.L.); pengyuhaopyh@163.com (Y.P.); 2 Guangdong Research Center on Reproductive Control and Breeding Technology of Indigenous Valuable Fish Species, Guangdong Ocean University, Zhanjiang 524088, China; 3 Guangdong Provincial Engineering Laboratory for Mariculture Organism Breeding, Zhanjiang 524088, China; 4 Guangdong Provincial Key Laboratory of Pathogenic Biology and Epidemiology for Aquatic Economic Animals, Zhanjiang 524088, China

**Keywords:** anorexigenic, food intake, neuropeptide Y, orexigenic, teleost fish

## Abstract

Neuropeptide Y family (NPY) is a potent orexigenic peptide and pancreatic polypeptide family comprising neuropeptide Y (Npy), peptide YYa (Pyya), and peptide YYb (Pyyb), which was previously known as peptide Y (PY), and tetrapod pancreatic polypeptide (PP), but has not been exhaustively documented in fish. Nonetheless, *Npy* and *Pyy* to date have been the key focus of countless research studies categorizing their copious characteristics in the body, which, among other things, include the mechanism of feeding behavior, cortical neural activity, heart activity, and the regulation of emotions in teleost. In this review, we focused on the role of neuropeptide Y gene (*Npy*) and peptide YY gene (*Pyy*) in teleost food intake. Feeding is essential in fish to ensure growth and perpetuation, being indispensable in the aquaculture settings where growth is prioritized. Therefore, a better understanding of the roles of these genes in food intake in teleost could help determine their feeding regime, regulation, growth, and development, which will possibly be fundamental in fish culture.

## 1. Introduction

Fish, the most distinguished group among vertebrates (over 30,000 species) [1], comprising approximately 95% teleost species, are the organisms most attracted for the study of the evolution of appetite-regulating systems in vertebrates [2,3]. This is due to their greater diversity in anatomy, ecology, behavior, and genomics [4,5,6]. Feeding is an important physiological activity in fish, necessary to ensure growth and survival. Food is one of the most authoritative external signals that can arouse fish feeding behavior and growth [7,8]. However, its availability and composition wield a precarious mechanism principally acting on the hormones responsible for their endocrine regulation [9]. Feeding is the outcome of an adjustment between starvation, appetite, and satiation. Starvation is the physiological requirement for food and comprises a solid stimulation to feeding conduct, including searching for food and feeding. Satiation is the physiological and mental sense of “fullness” that happens after eating, while appetite, on the other hand, is the longing to eat, which is ordinarily related to tactile (locate, scent, taste) perceptiveness of food [10].

In fish and other vertebrates, several hormones control feeding, including those produced by the brain and marginal organs [11,12]. It is known to be an intricate process that is vital to stimulate the survival of animals and the capacity to stay affected by elements, such as light, temperature, reproduction, and even the sort of food consumed. Food intake is governed by a fundamental and outlying nourishing scheme allied by a grid of peptides and hormones that control the sensitivity to eating and satiation [13,14,15]. 

Appetite and body weight control are multifaceted processes that involve extensive interactions between the brain and peripheral signals in all vertebrates. The brain (hypothalamus) produces key factors that either stimulate (orexigenic) or inhibit (anorexigenic) food intake in vertebrates (teleost) [12]. Knowledge about neuroendocrine control on food intake and regulation, including neuropeptide Y (*Npy*) and peptide YY (*Pyy*), explicitly concerning their roles, has significantly improved nowadays. Current studies have indicated that these peptides have impacts on the feeding behavior in vertebrates (teleost) [12,14,16,17,18] either as an orexigenic or anorexigenic factor.

Neuropeptide Y family (NPY) is a potent orexigenic peptide and pancreatic polypeptide family comprising neuropeptide Y (Npy), peptide YYa (Pyya), and peptide YYb (Pyyb), which was previously called peptide Y (PY), and tetrapod pancreatic polypeptide (PP) [19,20,21]. Both *Npy* and *Pyy* (Pyya and Pyyb) contain highly conserved amino acid sequences [19], whereas PP has evolved more rapidly but has fully not been recognized in fish [22,23,24]. Nonetheless, two peptides (Npy and Pyy) to date have been key focus of countless research articles, categorizing their copious characteristics in the body, which, among other things, include the mechanism of feeding behavior (one playing the role of food inducer, while the other is a food inhibiter), cortical neural activity, heart activity, and the regulation of emotions [24,25,26,27,28,29,30,31,32].

Among all the roles that the neuropeptide Y family genes play in fish, this review focuses significantly on their functions in feeding. Feeding is essential to ensure growth and perpetuation in living organisms, including fish. Therefore, a better understanding of the roles of these genes and their effects on food intake in teleost could help determine their feeding regime, regulation, growth, and development, which will possibly be fundamental in fish culture. Herein, we examined the current studies on the roles of *Npy* and *Pyy* in the regulation of food intake in teleost, as well as information gaps and future research directions.

### 1.1. Fundamental Characteristics of Npy and Pyy in Teleost

Npy, a peptide with 36 amino acid (AA) residues that was first isolated from porcine brain [33], is one of the most highly conserved neuropeptides in vertebrates [34,35]. It is a 36-amino-acid peptide produced from a 96-amino-acid pre-pro-peptide containing a 28-amino-acid N-terminal signal peptide and a 32-amino-acid C-terminal extension. Two classes of *Npy* (Npya and Npyb) have been discovered in some teleosts. However, teleosts such as the goldfish (*Carassius auratus*) and zebrafish (*Danio rerio*) have only Npya [20,36]. *Npy* is known to be chiefly secreted by the hypothalamus’ neurosecretory cells and is secreted in response to hunger [19,37]. Its primary function as a signaling factor is to regulate a variety of biological processes such as food intake, daily fixed cycle, neuroendocrine functions, and glucose homeostasis [38].

Pyy, conversely, belongs to a potent orexigenic peptide and pancreatic polypeptide (PP) family [19,20,39]. It is secreted from pancreatic endocrine cells (PP cells). Pyy has two endogenous forms: the full-length Pyy1–36 and the abridged form Pyy3–36 [40,41,42]. Both Pyy1–36 and Pyy3–36 can subdue appetite and food intake and delay gastric emptying [43]. *Pyy*, as an anorexigenic signal in teleost, is known to be a brain–gut peptide with its principal role as a satiety hint [27,29,44,45,46,47]. It has been approximated to be 70% homologous to Npy and PP; the configuration of amino acids for this peptide is also highly well-maintained within species [48]. It is secreted from the endocrine cells of the ileum and colon and functions by inhibiting *Npy* neurons in fish [49,50].

### 1.2. Expression of Npy and Pyy in Teleost

The Neuropeptide Y gene (Npy) has shown its expression in many tissues of several teleosts. It expresses itself in the central nervous system, intestine, liver, spleen, skeletal muscle, and fat tissue of several fish species, such as zebrafish (*Danio rerio*), goldfish (*Carassius auratus*), Atlantic salmon (*Salmo salar*), catfish (*Ictalurus punctatus*), and tilapia (*Oreochromis* sp.) [51,52,53]. It has also been detected in some teleost eyes but with little information [54,55]. 

Peptide YY mRNA (*Pyy*) also has shown expression in the kidney, gills, and within the brain—specifically the hypothalamus and pituitary—in some teleosts, including Atlantic salmon (*Salmo salar*), zebrafish (*Danio rerio*), goldfish (*Carassius auratus*), and Japanese eel (*Anguilla japonica*) [29,51,56,57]. It has also been identified in the gastrointestinal tract (GIT) of teleosts at the apparent highest levels in the stomach, pyloric caeca, foregut, and liver, and at lower levels in the hindgut [28,29,44,46,47,58]. 

In addition to this, our unpublished research study on the spotted scat (*Scatophagus argus*) revealed the expression of the *Npy* and *Pyy* genes in the central nervous system (brain) and some peripheral tissues (Assan et al., unpublished data).

### 1.3. Receptors of the Neuropeptide Y Family in Teleost

There is wide-ranging information about NPY and their receptors and neuro-endocrinological functions in non-mammalian vertebrates [22,23,24]. NPY is more intricate in teleost fish as compared to mammals. The NPY receptors of fish are articulated in the brain but can also be sited in marginal tissues, including the eye and intestine [59,60,61]. It is projected that the development of NPY peptides comprises the replication of a distinct congenital gene in an early vertebrate before the origination of vertebrates that possess jaws, ensuing in *Npy* and *Pyy* [62]. 

To date, seven types of receptors of NPY, known as the “Y receptors”, have been identified: Y1, Y2, Y4, Y5, Y7, Y6, and Y8, of which five are present in mammals (Y1, Y2, Y4, Y5, and Y6) [20,63,64]. All of these belong to the G-protein-coupled receptor; they have been categorized into two groups: the Y1-Y4-Y6-Y8 and the Y2-Y7 groups (Matsuda et al., 2012). These receptors are chiefly expressed in neural tissue and receptors in instinctual organs (such as the kidney and intestine), respectively [63,64,65]. NPY receptors vary in their ligand affinity profiles, of which Y1, Y2, and Y5 have a high affinity for *Npy* [66,67]. According to Dumont et al. [68], Pyy commits to all of the Y receptors, but the utmost affinity is seen for the Y2 receptor. Out of these seven NPY receptors, Y1 and Y2 have been consistently associated with the regulation of appetite with *Npy* [66,69]. 

According to Salaneck et al. [70] and Sundström et al. [20], Y1, Y2, Y4 (Ya), Y7, Y8a (Yc), and Y8b (Yb) have been traced in teleosts. They are pancreatic kinfolk polypeptides activated and characterized by NPY. The Y1 and Y5 receptors have been acknowledged to be intricate in the statute of orexins in mammals and fish [71]. The Y1 receptor-signaling pathway of *Npy* is known to stimulate food intake in teleost fish such as goldfish (*Carassius auratus*) and zebrafish (*Danio rerio*) [24,72,73]. The Y1 and Y2 receptor genes are comprehensively expressed in several expanses of the brain, but the expression of Y4 and Y5 receptor genes is constrained to precise loci involved in the directive of appetite, circadian rhythm, and apprehension [74]. There is a greater need for additional research that would help clarify the efficient rapport between the receptors of NPY, particularly that of fish.

## 2. Neuropeptide Y—Its Role as Feed Regulator in Teleost

The participation of neuropeptide Y in feeding behavior was proven in 1984 by three groups of researchers who indicated that the food intake of rats improved intensely when *Npy* was administered to their brains [75,76,77], and it is known to be one of the most abundant and effective orexigenic peptides found in the brain [78]. It plays a fundamental role in the regulation of food intake and the heftiness of the body inside the hypothalamus. The feeding-arousing influence of *Npy* is estimated to be about 500 times more effective on a molar basis than norepinephrine [79]. *Npy* stimulates appetite and consummatory actions under a range of circumstances [80]. 

Hunger-stimulating hormones in teleosts, for example, *Npy* [9,81] and *orexin* [45,82], have generally been known to exhibit changes before and during eating (higher or increased expressions) as well as after eating (lower or reduced expressions), which signifies them as food intake inducers. A study on chinook salmon (*Oncorhynchus tshawytscha*) and coho salmon (*Oncorhynchus kisutch*) (one of the foremost studies indicating the role of *Npy* in regulating food intake in fish) revealed, using in situ hybridization (ISH), that *Npy*-like mRNA signal zones were more common in starved fish than in fed fish [83]. The feeding regimen in teleost is firmly controlled by peptide diffusion contained by the hypothalamus [84]. In fish, the intake of feed is regulated by several hormones that are formed together by the brain and peripheral tissues, as stated earlier in this review. Feeding conduct inconsistencies and appetite recurrently transpire over the modulation of the gene expression and/or action of these appetite-regulating hormones [15]. *Npy* happens to be a hypothetically valuable regulator of fish feeding and development [85,86]. Numerous vital and outlying appetite regulators are affected by a lone meal, displaying prior feeding vacillations in their expression and/or secretion levels. Brain hormones demonstrating such changes in teleost include *Npy* [9,81], which is of interest in this review; orexin [45,82,87,88]; cocaine- and amphetamine-regulated transcript (*CART*) [18,87,88,89]; and nesfatin-1 [90].

Food deprivation is one of the most significant aspects that causes an increase in the expression of hypothalamic *Npy*. The discharge of *Npy* is boosted proximately earlier to the inception of feeding and progressively reduces as food ingestion continues [91]. The *Npy* gene has been identified in several teleost species, including goldfish (*Carassius auratus*) [92], zebrafish (*Danio rerio*) [24], and Ya-fish (*Schizothorax prenanti*) [58], which is associated with food intake. Intracerebroventricular (ICV) administration of *Npy* stimulated food intake in goldfish (*Carassius auratus*) [92] and also in zebrafish (*Danio rerio*) [24]. After long-term fasting, the expression of *Npy* was remarkably augmented in Ya-fish (*Schizothorax prenanti)*; its expression in the brain lessened after a meal and subsequently increased after fasting for two weeks [58]. *Npy* mRNA expression was steadily upregulated throughout the hunger period in the brain of blunt snout bream (*Megalobrama amblycephala*), and there were noticeable differences in the brain tissue among the fed group and the unfed group after starvation [93]. *Npy* in the brain increased after fasting and decreased after refeeding, showing that it functioned as an orexigenic factor to boost food intake [93], confirming how *Npy* in fish stimulates appetite. Moreover, improved *Npy* echelons have also been testified in the prosencephalon of Atlantic cod (*Gadus morhua*) in the course of mealtimes, which decreased after 2 h [94]. Kehoe and Volkoff reported that, despite *Npy* being an appetite stimulator in Atlantic cod, starvation did not affect the expression of *Npy* [94]. 

The regulation of growth hormone (GH) secretion is well understood to be controlled by a complex neuroendocrine control system, especially by the functional interplay of two hypothalamic hypophysiotropic hormones, GH-releasing hormone (GHRH) and somatostatin (SS), which exert stimulatory and inhibitory influences on the somatotrope, respectively [95]. With the help of in vitro and in vivo assays, *Npy* treatments have been revealed to stimulate growth hormone (GH) release in goldfish (*Carassius auratus*) [96] and tilapia (*Oreochromis mossambicus*) [97,98], respectively, as well as in orange-spotted grouper (*Epinephelus coioides*) [99] and catfish (*Clarias garipinus*) [100], leading to the hypothesis that *Npy* tends to be active in the regulation of pituitary GH in teleost.

Nonetheless, research by Wang et al. [85] revealed a relatively complex regulation of *Npy* mRNA expression in olive flounder (*Paralichthys olivaceus*). Their study revealed that *Npy* expression levels in the brain displayed an upsurge in the short time before and a decline after food intake. Furthermore, its expression diminished expressively in the three hours group after feeding, likened to that in the one-hour group before feeding, which confirms the result of Narnaware [52]. The research by Wang et al. [85] regarding the pre-prandial upsurges of the expression of the *Npy* gene before the scheduled feeding period presumed the responsiveness of hunger or how the flounder is expecting to eat or be fed (*Paralichthys olivaceus*). On the other hand, this same research with regard to the fasting experiment of the olive flounder (*Paralichthys olivaceus*) revealed that there was an 81.7% and 91.7% decrease in *Npy* in 24- and 48-h fasted olive flounders (*Paralichthys olivaceus*) respectively (Wang et al., 2015). This study was compared to that on goldfish (*Carassius auratus*), where fasting after 72 h led to a significant and time-related increase in *Npy* expression levels [85]. This helps us to understand that the physiological characteristics of *Npy* in teleost can also differ, and this could be attributed to the evolution of various mechanisms among taxa and species, likewise being influenced by innumerable extrinsic and intrinsic factors [85,88].

## 3. Peptide YY—Its Role as Feed Regulator in Teleost

**Figure 1:**
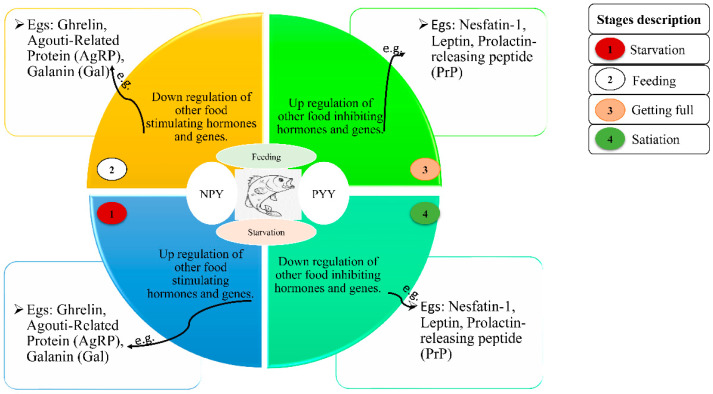
Effects of the *Npy* and *Pyy* genes on teleost food intake and their interplay with other central and peripheral endocrine factors or hormones to regulate growth hormone (GH).

Several recent studies have demonstrated that *Pyy* has an appetite-regulating effect on fish similar to that described in mammals [27,45,46]. *Pyy* is known to function as an anorexigenic (loss of appetite) indicator in mammals [101,102]. Studies have indicated that the *Pyy* gene (either *Pyya* or *Pyyb*) has been isolated and recognized in some fish species, including Atlantic salmon (*Salmo salar*) [51], goldfish (*Carassius auratus*) [29], Ya-fish (*Schizothorax prenanti*) [103], and Japanese eel (*Anguilla japonica*) [56]. Additionally, several studies have recounted that the expression of *Pyy* is affected by fasting in some fish. For example, fasting radically decreased *Pyy* mRNA expression in the brain of goldfish (*Carassius auratus*) [29] and the hypothalamus of Ya-fish (*Schizothorax prenanti*) [103], which signifies that *Pyy* plays an anorexic role in fish. A research study revealed that the expression of *Pyy* mRNA in the hypothalamus of *Schizothorax davidi* was expressly higher at +1 and +3 h post-feeding in the fed groups. *Pyy* mRNA expression levels emphatically declined in the hypothalamus of unfed fish compared with that of fish fed daily for 1, 3, 5, and 7 days [104]. According to these same researchers [104], the manifestation levels of *Pyy* augmented abruptly after refeeding after 9 days, signifying that it functions as a satiety factor in *Schizothorax davidi* and some other teleosts [28,29,45]. Food deprivation in some teleosts, such as goldfish (*Carassius auratus*), [29], Ya-fish (*Schizothorax prenanti*) [103], and red-bellied piranha (*Pygocentrus nattereri*) [44], caused a reduction in *Pyy* mRNA expression. Contrasting results have been reported while analyzing intestinal sections from fed vs. fasted fish. Fasting lessened the expression in piranha (*Pygocentrus nattereri*) [44], augmented it in yellowtail (*Seriola quinqueradiata*) [46], and did not cause a change in Atlantic salmon (*Salmo salar*) [51]. *Pyy* mRNA expression was also amplified in the GIT of grass carp post-feeding [28]. Equally, in Mexican blind cavefish (*Astyanax fasciatus mexicanus*), starvation did not disturb the expression of *Pyy* mRNA [45]. Comparable findings were also testified in Atlantic salmon (*Salmo salar*) [51] and red-bellied piranha (*Pygocentrus nattereri*) [44]. These clarifications suggest that the supervisory mechanism and response of *Pyy* to starvation and/or feeding might be species-specific [44,103], which could be attributed to the difference between their feeding habits, physiological processes, and digestive tract, as well as their being influenced by countless extrinsic and intrinsic factors. Additionally, the role that *Pyy* plays in teleost appetite control, however, seems to be vague and may be age-specific and/or particular to species, as previous results have indicated [51,105], among other reasons such as time and the tissue in which the *Pyy* gene was expressed [44]. Hence, there is a need for more research to confirm the difference. The figure below (Figure 1) describes how the *Npy* and *Pyy* genes work in fish alongside other food-stimulating and inducing hormones or genes and other endocrine factors. Table 1 shows a list of teleosts and their response to appetite-regulating hormones.

Starvation or fasting in fish habitually stimulates the upregulation of orexigenic factors and downregulation of anorexigenic factors, respectively, in teleost, while feeding deactivates the expression of orexigenic factors and upregulates anorexigenic factors in teleost. As the mRNA expression of *Npy* increases, it stimulates the secretion of GH and aids in fish growth. In other words, both *Npy* and GH work hand-in-hand to regulate growth [92,94,100,106,107,108,109].

## 4. Key Recognized Appetite-Regulating Endocrine Factors in Teleost 

Food consumption is ultimately controlled by the central feeding hubs of the brain, which obtain and process information from endocrine indications from both the brain and peripheral tissues [32]. Apart from *Npy* and *Pyy*, other known central and peripheral endocrine factors play key roles in regulating food intake in fish, either as an orexigenic or anorexigenic factor. The table below gives a list of some other appetite-regulating hormones in teleosts (Table 2).

### The Cause of the Unusual Role of Appetite-Regulating Endocrine Factors in Teleost (Precise Features Habituating Fish Food Intake)

Controlling food intake and energy metabolism is critical for an organism’s growth and survival. These processes ensure that energy resources are allocated optimally to cover basic metabolic and immune system maintenance, the cost of foraging and other daily activities, somatic development, reproductive expenditure, and adequate energy reserves to survive periods of low food availability [125]. External factors, as well as internal factors, influence food consumption [126], thereby influencing the basic endocrine mechanisms of the organism. The basic endocrine mechanisms that influence feeding, as in mammals, appear to be well-maintained between vertebrates such as teleost fish, but in terms of specificity in species, some complex regulatory mechanisms may exist in their appetite regulation [32]. The build-up and role of appetite-regulating hormones in fish, although similar to those of other vertebrates, showcase some key dissimilarities and might be based on the fish species considered. To date, the gesticulating mechanisms that regulate the consumption of food in fish and the reason why some hormones or genes play unusual roles in regulating appetite in teleosts are still unclear. As a consequence, the optimization of these hormones and food intake has become debatable, making it relatively complex to draw conclusions. Therefore, a better understanding of the endocrine mechanisms regulating feeding and fish growth might unravel the mystery behind some of these appetite-controlling hormones’ unusual roles.

As it’s known, appetite regulators and the expression levels of orexigenic factors usually increase before or during a meal (e.g., *Npy* [92]), whereas the expression levels of anorexigenic factors decrease after feeding (e.g., *Pyy* [44,103]). However, due to both intrinsic and extrinsic factors, these appetite regulators either play opposite roles or do not affect fish at appropriate times. It has been identified that in the intake of food in teleost fish, intrinsic factors including metabolic signals/energy reserves, during ontogeny; thus, from the time of fertilization of the egg to its maturity stage, gender and reproductive status, as well as genetic influence (different genetic or phenotypic makeup), affect the roles of appetite-regulating hormones [3]. 

There is a close relationship between feeding, gender, and reproductive parameters among fish, and there are previous reports on sex-specific differences in feeding behavior between fish species [10]. An example given here from Green et al. revealed that compared to territorial female cunners (*Tautogolabrus adspersus*), the males feed less often and have different diets during the spawning period [127]. With regard to sex-specific differences in levels of hormones regulating appetite in fish, research studies from Parhar et al. and Sakata et al. revealed that the level of gastrin ghrelin mRNA in female tilapia (*Oreochromis niloticus*) is higher compared to that in the males [128]. In female rainbow trout (*Oncorhynchus mykiss*), the number of ghrelin cells per unit area in the stomach is higher than that in the males [129]. Alternatively, reproductive parameters are known to affect food intake and appetite-regulating hormone expression in fish [10]. Research studies on European eel (*Anguilla anguilla*) [130], Atlantic salmon [131], and Atlantic cod (*Gadus morhua*) [132,133] showed that during the spawning and spawning migration periods, these fish eat very little as compared to the resting periods or when migrating to spawn. Additionally, in the domino damselfish (*Dascyllus albisella*), males have a smaller stomach content as compared to similar-sized females, since their time spent on feeding during courtship and nest safeguarding is known to reduce [134].

Additionally, in vertebrates (teleost), it has been discovered that appetite-regulating hormones affect their reproductive events, while contrarily, reproductive hormones can influence food intake and mRNA expression of appetite-regulating hormones [10]. Giving respective examples, *Npy* stimulated the release of gonadotropins and gonadotropin-releasing hormone (GnRH) in several fish species [135]. *Npy* mRNA expression in brain has been found to be twice as high in adult Brazilian flounder (*Paralichthys orbignyanus*) than that of the juvenile fish [136], signifying that *Npy* may have a significant role in the sexual growth and reproductive procedures in fish. On the other hand, a noteworthy decrease in feeding was exposed in seabass (*Dicentrarchus labrax*) after they were treated with testosterone and estrogen implants [137]. Additionally, central injection with GnRH in goldfish (*Carassius auratus*) triggered a decrease in its feeding, which, in one way or another, related to the downregulation of the expression of orexin, an appetite-regulating hormone in the brain [117]. Going forward, reproductive-stage changes correlated with changes in *Npy* immunoreactivity in the forebrain of catfish (*Clarias batrachus*), which aids in regulating food intake [138]. All of these results give a huge confirmation that the roles of appetite-regulating hormones in teleost are directly or indirectly affected by these intrinsic factors. 

The disparities in numerous extrinsic factors, including temperature, hypoxia, light regime and wavelength, photoperiod, salinity, among others, have a higher influence on the feeding conduct of teleost fish [15,139] and have also been identified to stimulate fluctuations in swimming activity and fish growth [140]. Some of these influences change in a recurring mode, and this, in one way or another, affects feeding either directly through periodic and 24-hourly rhythms [141] or indirectly through rhythms in endocrine systems [142,143]. Two of the most significant ecological factors, temperature and photoperiod, have been known to influence the intake of food and appetite regulation in fish. However, the distinct effects of these parameters under natural conditions might be difficult to distinguish, as fish are subjected to periodic cycles in which both factors vary [3]. As reported by Kehoe and Volkoff in Atlantic cod (*Gadus morhua*), the specific endocrine mechanisms behind these changes and the role of appetite-regulating hormones with regard to temperature and photoperiod are still unclear [144]. Their research was based on environmental temperature influence on feeding and the expression levels of one appetite-inducing hormone, neuropeptide Y, and one appetite-inhibiting hormone, cocaine and amphetamine-regulated transcript. It revealed that Atlantic cod (*Gadus morhua*) adjusted to 2 °C for 4 weeks had a decreased food intake and higher *CART* brain mRNA levels compared to fish adjusted to 11 and 15 °C. There were no differences in *Npy* mRNA expression between the three groups. Their results suggest that low temperatures inhibit food intake in Atlantic cod (*Gadus morhua*), and this inhibition is in part mediated by an increase in *CART* expression but does not involve changes in *Npy* expression. This implies that *CART*, but not *Npy*, may be involved in facilitating temperature-induced changes in appetite in fish, which still draws us back to the fact that the biological make-up of fish in terms of species specificity, their feeding habits, physiological processes, and digestive tract, among others, has a firm role to play in food intake and hormones regulating their appetite.

## 5. Conclusions

In summary, the two appetite-regulating hormones *Npy* and *Pyy* have a widespread mRNA distribution in numerous teleost fish. They have attracted significant interest in recent years due to the specific role each plays in the regulation of feeding and are of great importance to how teleost fish react to food intake, either as hunger or satiety signaling. We inferred from the information gathered that *Npy* acts as an orexigenic factor in teleost whilst *Pyy* acts as an anorexigenic factor in fish in almost all cases. The general expected roles of *Npy* and *Pyy* in some teleosts have been misplaced, in the sense that there are countless factors that in some way influence the roles of these genes, making it quite difficult to conclude that the expression or role of *Npy* and *Pyy* affects all teleost similarly. Interestingly, starvation duration influences appetite-regulating hormones differently in different fish. Hence, comprehensive research studies should be conducted on these appetite-regulating genes and their unusual roles in regulating food intake, taking into consideration these two key points: firstly, how these factors influence the general expected roles of *Npy* and *Pyy* in teleost fish, and secondly, the ways in which these factors could help us understand more about the roles of *Npy* and *Pyy* in teleost fish.

## Figures and Tables

**Table 1:** List of teleosts and their response to appetite-regulating hormones.

Appetite-Regulating Hormones	Treatment	Duration	Gene Regulation	Fish	Reference
***Neuropeptide Y*** *(Npy)*	Starvation	3 weeks	Upregulation	Chinook salmon (*Oncorhynchus tshawytscha*)	[83]
				Coho salmon (*Oncorhynchus kisutch*)	
		3 days to 1 week		Goldfish (*Carassius auratus*)	[52]
		1 week		Zebrafish (*Danio rerio*)	[24]
		2 weeks		Ya-fish (*Schizothorax prenanti*)	[58]
		1 day		Blunt snout bream (*Megalobrama amblycephala*)	[93]
		1 week	No effect of *Npy* expression	Atlantic cod (*Gadus morhua*)	[94]
		1–2 days	Downregulation	Olive flounder (*Paralichthys olivaceus*)	[85]
***Peptide YY*** *(Pyy)*	Feeding and or refeeding	9 days	Upregulation	*Schizothorax davidi*	[104]
	Refeeding after fasting	3 days		Yellowtail (*Seriola quinqueradiata*)	[46]
		1 week	Upregulation, as compared to fish fed for the whole week	Goldfish (*Carassius auratus*)	[29]
	Starvation	6 days	No effect of *Pyy* expression	Atlantic salmon (*Salmo salar*)	[51]
		10 days	No effect of *Pyy* expression or no significant difference between fed and fasted fish	Mexican blind cavefish (*Astyanax fasciatus mexicanus*)	[45]
		9 days	Downregulation; increased after refeeding	Ya-fish (*Schizothorax prenanti*)	[103]
		1 week	Downregulation	Red-bellied piranha (*Pygocentrus nattereri*)	[44]

**Table 2:** Other known appetite-regulating hormones in teleosts.

Appetite-Regulating Hormone	Orexigenic/Anorexigenic Actions	Tissues	Reference
Ghrelin	Orexigenic	Gastrointestinal tract	[110]
Growth hormone (GH)	Orexigenic	Pituitary	[111,112]
Calcitonin gene-related peptide	Orexigenic	Brain	[113,114]
Prolactin-releasing peptide (PrP)	Anorexigenic	Brain	[115]
Leptin	Anorexigenic	Liver	[116]
Gonadotropin-releasing hormone (GrH)	Anorexigenic	Brain	[117]
Agouti-related protein (AgRP)	Orexigenic	Brain	[88,118]
Proopiomelanocortin (POMC)	Anorexigenic	Pituitary	[88]
Cocaine- and amphetamine-regulated transcript (CART)	Anorexigenic	Brain, pituitary	[107]
Orexins	Orexigenic	Hypothalamus	[88,119]
Nesfatin-1	Anorexigenic	Hypothalamus	[120]
Galanin (Gal)	Orexigenic	Gastrointestinal tract and the brain	[121]
Cholecystokinin (CCK)	Anorexigenic	Gastrointestinal tract and the brain	[120,122]
Glucagon-like peptide-1	Anorexigenic	Brain and intestine	[9,123,124]
